# Statistical modeling to analyze factors affecting the middle-income trap in Indonesia using panel data regression

**DOI:** 10.1016/j.mex.2023.102379

**Published:** 2023-09-15

**Authors:** Vita Ratnasari, Salsabila Hidayatul Audha, Andrea Tri Rian Dani

**Affiliations:** a Department of Statistics, Faculty of Science and Data Analytics, Institut Teknologi Sepuluh Nopember, Surabaya, Indonesia; b Statistics Study Program, Department of Mathematics, Faculty of Mathematics and Natural Sciences, Mulawarman University, Samarinda, Indonesia

**Keywords:** COVID-19, Economy, Middle-income trap, Panel data, Regression, Panel Data Regression

## Abstract

The Middle-Income Trap (MIT) refers to the situation in which a country's per capita income can reach middle-class levels but remains there for years, making it difficult to move up to a higher income level. Indonesia was declared trapped in MIT in 2014, and in 2019, it was added to the group of upper-middle-income (UM) countries. However, due to COVID-19, it returned to the lower-middle income (UM) group in 2020. Previous studies inspired to see the existence of provinces in MIT Indonesia, so an MIT analysis was carried out for each province. The analysis aims to determine the characteristics of MIT in Indonesia and the factors that influence it and obtain a panel data regression model formed from MIT modeling. Analysis using panel data regression method. The panel data regression model was obtained based on panel data, namely data consisting of a combination of cross-section and time series data. The data used is GRDP per capita for each province in 2010 – 2020 published by the Central Bureau of Statistics (BPS). GRDP per capita data is used as an approach to calculating the MIT index. The results of the study, obtained three variables that have a significant effect on MIT, namely Life Expectancy, Gross Participation Rate, and Gross Fixed Capital Increase with an accuracy model of 97.65 %.

## Review Highlights

• Regression modeling with panel data.
• The observation units used are provinces in Indonesia based on inter-regional disaggregation variables in modeling the middle-income trap problem.

## Graphical abstract

**Figure:**
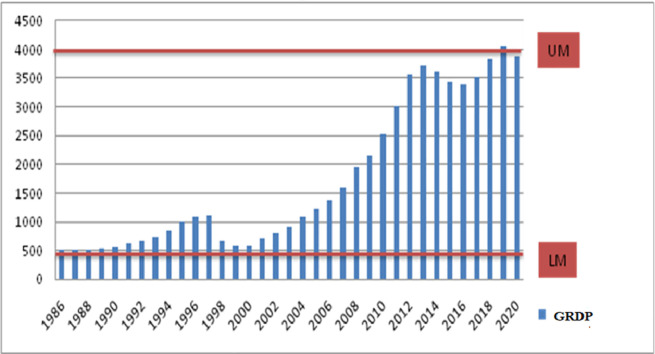

The characteristics of MIT in Indonesia. As mentioned in 2019, Indonesia entered the UM class but returned to LM status in 2020. The pandemic, which is suspected of having an impact on reducing Indonesia's income class, also occurred in Provinces in Indonesia, where most provinces experienced a decrease in GRDP per capita from the previous year during 2019–2020, and only ten provinces experienced an increase in per capita income.

Specifications table

**Table:** 

Subject area:	Mathematics and Statistics
More specific subject area:	Statistics; Regression; Economy
Name of the reviewed methodology:	Panel Data Regression
Keywords:	COVID-19, Economy, Middle-Income Trap, Panel Data Regression
Resource availability:	Middle-Income Trap (Y) and the predictors (X) from Badan Pusat Statistik and World Bank.
Review question:	How is the panel data regression model formed in the MIT modeling in Indonesia?

**Method details**

## Introduction

The term “Middle Income Trap” (MIT) was first used in 2004 [1]. It describes a country where the average income per person can reach middle-class levels but stays for a long time, and it is hard to move out [2], [3], [4]. It is assumed that this income stagnation occurs because of the inability of MIT countries to compete with high-income countries (which have an economy and quality of science that tends to be competitive). The spectrum of MIT discussion is relatively broad, starting from economic growth, the effects of social conditions, the effects of spatial development, to specialization,and innovation [4,5].

In 2014, Indonesia, a country with 34 provinces, had been stuck in MIT for more than 28 years and had a per capita income in the lower middle class [6]. In 2019, the Gross National Income (GNI) per person in Indonesia was $4050, which put it in the upper-middle income (UM) group of countries. Still, on July 1, 2020, Indonesia returned to the middle-low-income countries with a GNI per capita of US$ 3870. The economic contraction in Indonesia in 2020 due to COVID-19 has resulted in a correction to the level of welfare and Indonesia's status which has re-entered the lower middle-income country. In 2020, the growth rate of per capita Gross Regional Domestic Product (GRDP) at constant prices in almost all provinces in Indonesia shows a negative number. This indicates that the income class of people in Indonesia is decreasing due to the pandemic.

Based on Indonesia's MIT problems, which showed income stagnation, failure to transition to a high-income economy, and weak competitiveness [7], MIT became an essential topic for developing Indonesia to study and discuss. An analysis is needed to separate Indonesia from MIT. In the MIT analysis, it is closely related to the MIT conditions for each province and its transition from year to year, so one method that can be used is panel data regression [8].

The panel data regression model was obtained based on cross-section data and time series [9,10]. Data cross-section is data collected simultaneously with too many objects [11,12]. The thing here can be an individual as well as a group. At the same time, time series data is data that has been collected at a specific time against an object [13,14]. The estimation process in the panel data regression model has several models that can be formed due to differences in slope and intercept coefficients for each observation at a particular time. Because the slope and intercept coefficients are not the same, there are three ways to estimate panel data regression models: the Common Effect Model (CEM), the Fixed Effect Model (FEM), and the Random Effect Model (REM) [10,15]. Several studies have examined and developed panel data regression models, including [4,[16], [17], [18], [19], [20], [21], [22]]. This research focuses on analyzing what variables significantly affect MIT in Indonesia, which provinces have the most critical influence on MIT conditions in Indonesia, and their characteristics from year to year so that this Province can be maximized. Its potential is a reference for other provinces to affect conditions nationally, and Indonesia can be separated from MIT.

## Materials and methods

### A.  Panel regression

The panel data regression model is formed from panel data [23], which consists of observations on the same cross-sectional units or individuals over several periods (time series) [9]. The general panel data structure is shown in Table 1.

**Table 1:** Panel data structure.

Individuals	Time	Y	$X_{1}$	$X_{2}$	…	$X_{k}$
1	1	$Y_{11}$	$X_{111}$	$X_{211}$	…	$X_{k11}$
	2	$Y_{12}$	$X_{112}$	$X_{212}$	…	$X_{k12}$
	⋮	⋮	⋮	⋮	⋮	⋮
	T	$Y_{1T}$	$X_{11T}$	$X_{21T}$	…	$X_{k1T}$
2	1	$Y_{21}$	$X_{121}$	$X_{221}$	…	$X_{k21}$
	2	$Y_{22}$	$X_{122}$	$X_{222}$	…	$X_{k22}$
	⋮	⋮	⋮	⋮	⋮	⋮
	T	$Y_{2T}$	$X_{12T}$	$X_{22T}$	…	$X_{k2T}$
⋮	⋮	⋮	⋮	⋮	⋮	⋮
N	1	$Y_{N1}$	$X_{1N1}$	$X_{2N1}$	…	$X_{\text{kN}1}$
	2	$Y_{N2}$	$X_{1N2}$	$X_{2N2}$	…	$X_{\text{kN}2}$
	⋮	⋮	⋮	⋮	⋮	⋮
	T	$Y_{\text{NT}}$	$X_{1\text{NT}}$	$X_{2\text{NT}}$	…	$X_{\text{kNT}}$

The regression model equation using cross-section data can be written in Eq. (1).

(1) $$y_{i}=\alpha +X_{i}\beta +{ɛ}_{i}$$

With i=1,2,…,n, where n is the number of data cross sections. Then the regression model equation using time series data is written in Eq. (2).

(2) $$y_{t}=\alpha +X_{t}\beta +{ɛ}_{t}$$

With t=1,2,…,T, where T is the number of data time series. So that in general, the panel data regression model can be written in Eq. (3).

(3) $$y_{\mathrm{it}}=\alpha +X_{\mathrm{it}}^{\prime }\beta +{ɛ}_{\mathrm{it}}$$

Where:

$$y_{\mathrm{it}}=\left[\begin{array}{c} y_{i1}\\ y_{i2}\\ \vdots \\ y_{\mathrm{iT}} \end{array}\right]\;X_{\mathrm{it}}=\left[\begin{array}{cccc} X_{1i1} & X_{2i1} & \ldots & X_{\mathrm{ki}1}\\ X_{1i2} & X_{2i2} & \ldots & X_{\mathrm{ki}2}\\ \vdots & \vdots & \ddots & \vdots \\ X_{1\mathrm{iT}} & X_{2\mathrm{iT}} & \ldots & X_{\mathrm{kiT}} \end{array}\right]\;\beta =\left[\begin{array}{c} \beta_{1}\\ \beta_{2}\\ \vdots \\ \beta_{k} \end{array}\right]\;{ɛ}_{\mathrm{it}}=\left[\begin{array}{c} {ɛ}_{i1}\\ {ɛ}_{i2}\\ \vdots \\ {ɛ}_{\mathrm{iT}} \end{array}\right]$$

### B.  Regression model estimation

The Common Effect Model (CEM), the Fixed Effect Model (FEM), and the Random Effect Model (REM) are the three approaches that are typically utilized in the process of estimating panel data regression models [10]. Estimating parameters for CEM and FEM using Ordinary Least Squares (OLS) [24]. When estimating the REM parameter, used Generalized Least Squares, while the intercept FEM is expressed by a dummy variable (GLS).

(1) Common Effect Model (CEM)
  CEM is the same approach as estimating a multiple linear regression model. The principle of CEM is to regress all data combined without regard to time and individual effects [25]. In CEM, the intercept is stated to be constant or the same for each individual or at any time. The general Equation of the regression model from CEM is written in Eq. (4).
  (4) $$y_{it}=\beta_{0}+\beta_{1}X_{1it}+\beta_{2}X_{2it}+\ldots +\beta_{k}X_{kit}+\varepsilon_{it}$$
  Eq. (4) is written in matrix form as in Eq. (5).
  (5) y=Xβ+ɛ
(2) Fixed Effect Model (FEM)
  a.  FEM is a panel data regression estimation method that is used because it can accommodate the characteristics between individuals, which are adjusted through the intercept. The model can be estimated using dummy regression where each individual and time will be a dummy variable. Several types of FEM models are described as follows: a. FEM has a constant slope but variations in the intercept coefficient for each individual. This model ignores the effects of time, but there are different effects between individuals [25]. The formula with the following conditions is written in Eq. (6).
  (6) $$y_{it}=\alpha_{0}+\alpha_{1}D_{1i}+\ldots +\alpha_{N-1}D_{N-1i}+\beta_{1}X_{1it}+\ldots +\beta_{k}X_{kit}+\varepsilon_{it}$$
  with:
  $y_{it}$ : individual response variable, i th and t-period
  $\alpha_{0}$ : individual FEM model intercept
  $\alpha_{i}$ : the slope of the i th individual
  $\beta_{k}$ : the slope coefficient of the k-th predictor variable
  $D_{i}$ : dummy category for the i th individual
  $X_{kit}$ : the k-th predictor variable, the i th individual, and the t-period
  $\varepsilon_{it}$ : individual residual, i th and t-period
  b.  FEM with a constant slope, but there is a variation of the intercept coefficient at any time. The model ignores the effects on individuals, but there are effects at different times. The formula with the following conditions is written in Eq. (7).
  (7) $$y_{it}=\lambda_{0}+\lambda_{1}D_{1i}+\ldots +\lambda_{N-1}D_{N-1i}+\beta_{1}X_{1it}+\ldots +\beta_{k}X_{kit}+\varepsilon_{it}$$
  with:
  $\lambda_{0}$ : intercept the FEM time model
  $\lambda_{t}$ : the slope of the t-th time
  c.  FEM considers both effects, namely the individual effect and the effect of time. The formula with the following conditions is written in Eq. (8).
  (8) $$y_{it}=\alpha_{0}^{*}+\alpha_{i}+\lambda_{t}+\beta_{1}X_{1it}+\ldots +\beta_{k}X_{kit}+\varepsilon_{it}$$
  with:
  $\alpha_{0}^{*}$ : intercept the FEM time model
  $\alpha_{i}$ : the slope of the i th individual
  $\lambda_{t}$ : the slope of the t-th time
(3) Random Effect Model (REM)
  REM is the last approach in estimating panel data regression models. This model arises from FEM, which does not represent the actual model, so REM is used with the general Equation written in Eq. (9).
  (9) $$y_{\mathrm{it}}=\alpha_{i}+X_{\mathrm{it}}^{\prime }\beta +w_{\mathrm{it}}$$
  With $w_{\mathrm{it}}={ɛ}_{\mathrm{it}}+u_{i}$. ${ɛ}_{\mathrm{it}}$ is the combination of error components between cross-section and time series, and $u_{i}$ is the error component of the cross-section data.

### C.  Best model selection

Several tests were performed to determine the appropriate estimate of the panel data regression, including:

(1) Chow Test

The Chow test is a test to choose between the CEM and FEM models with the following hypothesis formulation:

$H_{0}$ : $\beta_{1}=\beta_{2}=\ldots =\beta_{k}=0$ (The suitable model is CEM)
$H_{1}$ : At least there is one $\beta_{k}\neq 0$, k=1,2,…,K (The suitable model is FEM)

Test statistics:

(10) $$F=\frac{\left(R_{LSDV}^{2}-R_{pooled}^{2}\right)/\left(N-1\right)}{\left(1-R_{LSDV}^{2}\right)/\left(NT-N-k\right)}$$

with:

$R_{LSDV}^{2}$ : coefficient of determination for the FEM model
$R_{pooled}^{2}$ : coefficient of determination for the CEM model
N  : number of individual units
T  : number of periods
k  : number of predictor variables

The critical area is rejected $H_{0}$ if $F>F_{\left(\alpha ;N-1;N(T-1)-k\right)}$.

(2) Hausman Test

The Hausman test was carried out after the Chow test concluded that the FEM model was the best. Furthermore, it is necessary to test to select the best model between the FEM and REM models with the following hypothesis formulation:

$H_{0}$ : $corr(X_{it},\varepsilon_{it})=0$ (The suitable model is REM)
$H_{1}$ : $corr(X_{it},\varepsilon_{it})\neq 0$ (The suitable model is FEM)

Test statistics:

(11) $$W={\left[b-\hat{\beta }\right]}^{{}^{\prime }}{\left[Var\left(b\right)-Var\left(\hat{\beta }\right)\right]}^{-1}\left[b-\hat{\beta }\right]$$

with:

b : beta matrix of the FEM model
$\hat{\beta }$ : beta matrix of the REM model

The critical area is rejected $H_{0}$ if $W>\chi_{\left(\alpha ;k-1\right)}^{2}$.

(3) Lagrange Multiplier (LM)

This test is used to choose between the CEM and REM estimation models under the following hypotheses:

$H_{0}$ : $\sigma_{1}^{2}=\sigma_{2}^{2}=\ldots \sigma_{N}^{2}=\sigma^{2}$ (The suitable model is CEM)
$H_{1}$ : $\sigma_{i}^{2}\neq \sigma^{2}$ (The suitable model is REM)

Test statistics:

(12) $$LM=\frac{NT}{2\left(T-1\right)}{\left[\frac{\sum_{i=1}^{N}{\left[\sum_{t=1}^{T}\varepsilon_{it}\right]}^{2}}{\sum_{i=1}^{N}\sum_{t=1}^{T}\varepsilon_{it}^{2}}-1\right]}^{2}$$

The critical area is rejected $H_{0}$ if $LM>\chi_{\left(\alpha ;N-1\right)}^{2}$.

### D.  Parameter significance test

Parameter significance testing was conducted to determine whether the predictor variable significantly affected the response variable [26,27].

(1) Simultaneous Test

A simultaneous parameter significance test is a parameter significance testing method that is carried out to find out how the influence of all predictor variables on the response variable with the following hypothesis formulation:

$H_{0}$ : $\beta_{1}=\beta_{2}=\ldots =\beta_{k}=0$
$H_{1}$ : At least there is one $\beta_{k}\neq 0$, k=1,2,…,K

The critical area is rejected $H_{0}$ if $F_{hit}>F_{\left(\alpha /2;k;NT-k-1\right)}$, with the test statistic in Eq. (13).

(13) $$F=\frac{MSR}{MSE}$$

(2) Partial Test

A partial parameter significance test is a method of testing the significance of parameters carried out to find out how each predictor variable influences the response variable with the following hypothesis formulation:

$H_{0}$ : $\beta_{k}=0$
$H_{1}$ : $\beta_{k}\neq 0$, k=1,2,…,K

The critical area is rejected $H_{0}$ if $t_{hit}>t_{\left(\alpha /2;NT-k-1\right)}$, with the test statistic in Eq. (14).

(14) $$t_{hit}=\frac{{\hat{\beta }}_{k}}{SE\left({\hat{\beta }}_{k}\right)}$$

### E.  Data sources

The data used in this study is secondary data obtained from the Badan Pusat Statistik and the World Bank. The data is from observations made for 33 Provinces in Indonesia from 2010 to 2020. The variables used include the response variable (Y) and predictor variable (X), detailed in Table 2.

**Table 2:** Description of study variables.

Variable	Notation	Explanation
Response	Y	Middle-Income Trap
Predictor	$X_{1}$	Average School Years
	$X_{2}$	Life expectancy
	$X_{3}$	Foreign Investment
	$X_{4}$	Gross Enrollment Rate
	$X_{5}$	Open Unemployment Rate
	$X_{6}$	Gross Fixed Capital Increase

### F.  Data analysis technique

Data analysis in this study consisted of the following steps:

(1) Perform data preprocessing. Convert GRDP per capita to US$ with the Atlas conversion method.
(2) Describe the characteristics and influencing factors of MIT in each Province of Indonesia.
(3) Check whether there is a multicollinearity assumption problem.
(4) In the process of carrying out Panel Data Regression Modeling, estimations were made concerning the parameters of the Common Effect Model (CEM), the Fixed Effect Model (FEM), and the Random Effect Model (REM) regression models.
(5) We select the best model with the Chow Test, Lagrange Multiplier, and Hausman.
(6) Perform a significance test of the regression model parameters from the results of selecting the best model.

## Main results

### A.  Data exploration

**Fig. 1:**
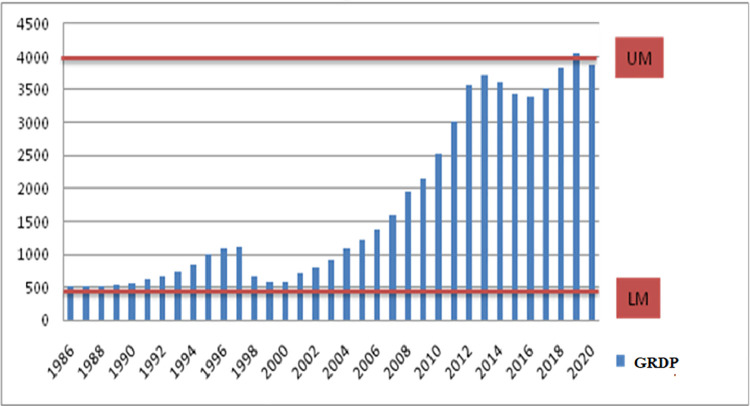
Indonesian income per capita.

The data exploration stage is the first step to finding out the characteristics of the research data. The benefits are that it will do reading and understanding the data easier. The characteristics of MIT in Indonesia are shown in Fig. 1.

Indonesia in 2020 is the 34th year of being stuck at MIT after starting in 1986, being in a Lower Middle-Income Country with a per capita GRDP of $3983. As previously mentioned 2019, Indonesia entered the UM class but returned to LM status in 2020. The pandemic, which is suspected of having an impact on reducing Indonesia's income class, also occurred in Provinces in Indonesia, where most provinces experienced a decrease in GRDP per capita from the previous year during 2019–2020, and only ten provinces experienced an increase in per capita income. The gap in per capita income between provinces in 2020 is relatively large, starting from the lowest, namely East Nusa Tenggara (US$ 1344), to the highest, namely DKI Jakarta, which is (US$ 18,217). DKI Jakarta has a per capita income almost 14 times the per capita income of East Nusa Tenggara. Conditions in 2020 have decreased compared to 2019. There is a difference in the three provinces that dropped the grade from UM in 2019 to LM in 2020. The three provinces that have decreased rates are Jambi, East Java, and Bali. This is why Indonesia dropped from Upper Middle (UM) Income to Lower Middle (LM) Income Country in 2020, allegedly closely related to the COVID-19 pandemic.

**Fig. 2:**
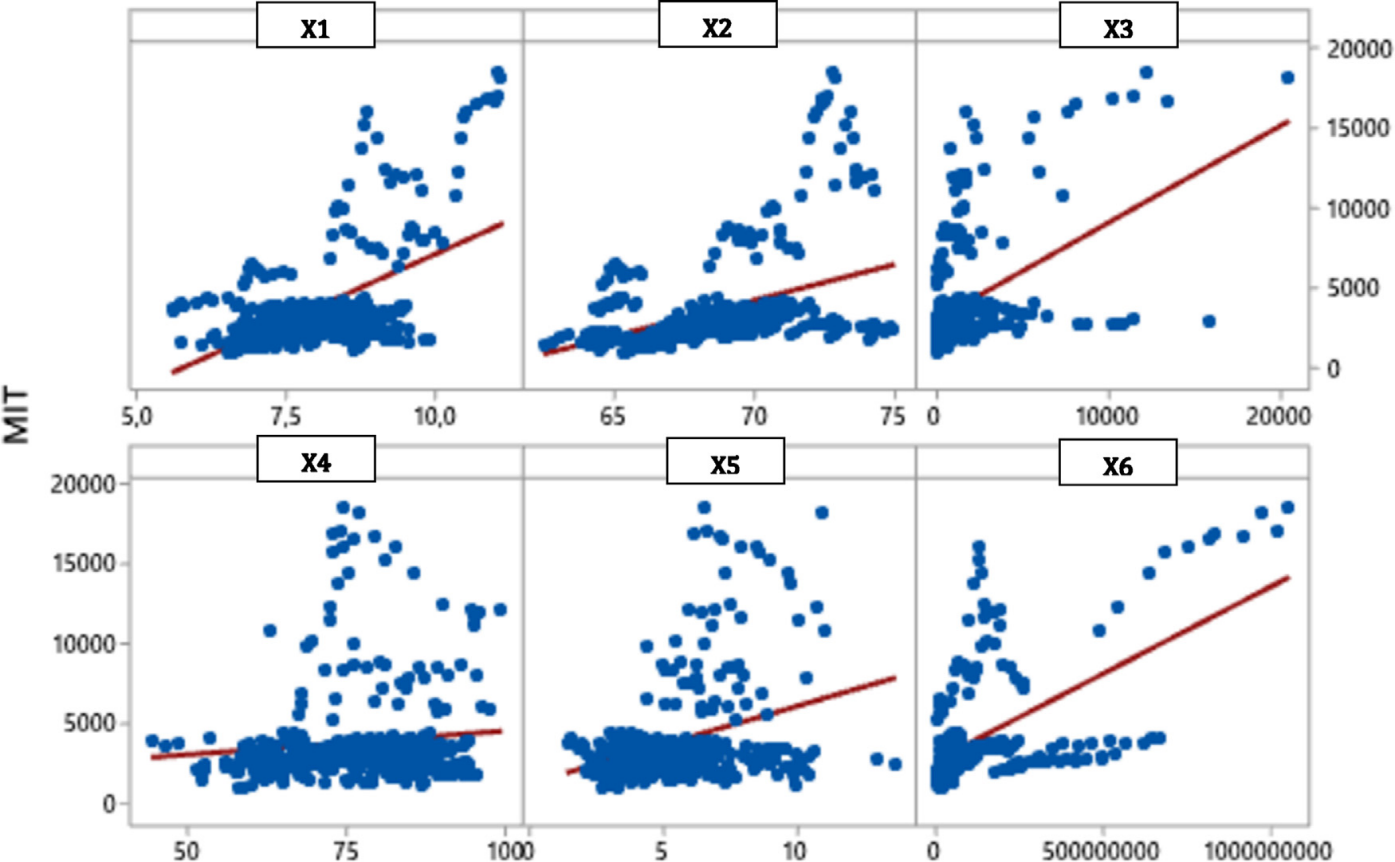
Scatter plot between response variable with predictor variables.

Based on Fig. 2, the relationship between the response variable Y with predictor variables $X_{1}$, $X_{2}$, $X_{3}$, $X_{4}$, $X_{5}$, and $X_{6}$ is positively related, which means that the greater the values of the predictor variable, the greater the probability that an area will be detached from MIT because the GRDP per capita will be more significant.

### B.  Multicollinearity detection

The multicollinearity assumption is critical before doing the modeling. In this study, the Variance Inflation Factor (VIF) value was used to determine whether there were cases of multicollinearity between predictor variables.

Table 3 shows that all predictor variables have a VIF value of less than 10, meaning there is no multicollinearity problem.

**Table 3:** VIF Values for each predictor variable.

Variable	$X_{1}$	$X_{2}$	$X_{3}$	$X_{4}$	$X_{5}$	$X_{6}$
VIF	2.25	1.48	3.36	1.59	1.41	3.46

### C.  Panel data regression analysis

The estimated MIT model will consist of three models: the Common Effect Model (CEM), the Fixed Effect Model (FEM), and the Random Effect Model (REM). The next step is to choose the best model from the estimated models. The initial step is the Chow test to determine the difference between the CEM and FEM models. Chow test calculations obtain test statistics 207.25 with a p-value of 0.000, then the decision $H_{0}$ is rejected, which means that the FEM model is the best.

Next, we will be continued with the Hausman test to choose between the FEM or REM models. It is known that the results of the Hausman test with test statistics obtained are 22.94 with a p-value of 0.000, then the decision $H_{0}$ is rejected, which means that the FEM model is the best. The Chow and Hausman test results conclude that the appropriate estimation method is FEM. Furthermore, the parameter significance test will be carried out simultaneously and partially. There are at least two models in the FEM model that will be tested because they have an R^2^ value of more than 70 %, namely individual FEM and individual-time FEM.

Simultaneous tests on the individual FEM model obtained the decision $H_{0}$ was rejected because a p-value of 0.000 means that at least one predictor variable significantly affects MIT. Next, a partial test will be carried out to determine the impact of each variable shown in Table 4.

**Table 4:** Individual FEM partial test.

Variable	|t|	P-value	Conclusion
$X_{1}$	−3.60	0.000	Significant
$X_{2}$	5.07	0.000	Significant
$X_{3}$	2.50	0.013	Significant
$X_{4}$	−1.98	0.049	Significant
$X_{5}$	−3.73	0.000	Significant
$X_{6}$	4.87	0.000	Significant

Based on Table 4, all predictor variables partially affect the response variable. The individual FEM models obtained are presented in Eq. (15).

(15) $$Y_{it}=-48,516.50+\alpha_{i}-807.82X_{1it}+866.27X_{2it}+0.08X_{3it}-12.14X_{4it}-111.78X_{5it}+4.06\;\times 10^{-6}X_{6it}$$

Based on the estimation results of the individual effect FEM model, the goodness of the model is obtained by 97.32 %, which means that the variables can explain the response variable, namely the MIT of 97.32 % $X_{1}$, $X_{2}$, $X_{3}$, $X_{4}$, $X_{5}$, and $X_{6}$.

Simultaneous testing on the individual-time FEM model obtained the decision H0 being rejected because a p-value of 0.000 was obtained, meaning that at least one predictor variable significantly affected MIT. Next, a partial test will be carried out to determine the impact of each variable shown in Table 5.

**Table 5:** Individual-Time FEM partial test.

Variable	|t|	P-value	Conclusion
$X_{1}$	−1.15	0.250	Not Significant
$X_{2}$	2.14	0.033	Significant
$X_{3}$	2.13	0.034	Significant
$X_{4}$	−2.34	0.020	Significant
$X_{5}$	−1.67	0.097	Not Significant
$X_{6}$	5.19	0.000	Significant

Based on the estimation results of the individual-time effect FEM models, two predictor variables were not significant, with a p-value greater than α of 0.05. Variables that are not significant will be removed from the model, and individual-time FEM model estimates will be carried out using significant variables.

Based on the estimation results of the individual-time effect FEM models without including the variables $X_{1}$ and $X_{5}$ into the model, the goodness of fit from the model is 97.67 %, which means that the MIT variable of 97.67 % can be explained by the variables $X_{2}$, $X_{3}$, $X_{4}$, and $X_{6}$. The model that has been obtained is then tested to determine whether it meets the assumptions that the residuals are identical, independent, and normally distributed. Some of these tests will be carried out on the best model obtained in the following.

(1) Individual FEM

All residual assumptions have not been met. It is shown in the results of the Glejser test that there are still six Provinces whose residuals are not identical because there are predictor variables that have a p-value of less than 0.05. Two Provinces experience autocorrelation, as seen on the ACF graph, where there is a lag outside the significance limit. The residuals are not normally distributed.

(2) Individual-Time FEM

The assumption of identical and normal residuals has not been fulfilled. It is shown in the results of the Glejser test that there are still 2 Provinces whose residuals are not similar because there are predictor variables that have a p-value of less than 0.05. There is no autocorrelation problem. The residuals are not normally distributed. Based on the analysis, it was concluded that the residual assumptions in the individual-effect FEM model and the individual-time effect FEM were not met. So solving and overcoming problems in the residual assumption test is necessary.

### D.  Panel data regression analysis with the white method

*Re*-estimating the panel data regression model using the White method (Heteroscedasticity-Corrected Standard Errors) to overcome violations of the residual assumptions. Simultaneous tests on the individual FEM model obtained the decision $H_{0}$ was rejected, which means that at least one predictor variable significantly affects MIT. Based on the estimation results of White's individual FEM model, two predictor variables were obtained that were not significant with a p-value greater than α of 0.05. Variables that are not significant will be removed from the model and estimated using significant variables by White's individual FEM model.

Based on the estimation results of White's individual FEM model without including the variables $X_{3}$ and $X_{5}$ into the model, the goodness of the model is 97.19 %, which means that the MIT variable of 97.19 % can be explained by the variables $X_{1}$, $X_{2}$, $X_{4}$, and $X_{6}$. White's individual FEM model obtained is presented in Eq. (16).

(16) $$Y_{it}=\alpha_{0}+\alpha_{i}-772.86X_{1it}+908.44X_{2it}+13.48X_{4it}+5.71\;\times 10^{-6}X_{6it}$$

Next, a significance test of the individual-time effect of the FEM model parameters will be carried out after being resolved. After re-estimating the model three times, the final model is obtained with the remaining three significant variables.

(17) $$Y_{it}=\alpha_{0}+\alpha_{i}+\lambda_{i}+402.67X_{2it}+18.30X_{4it}+5.49\;\times 10^{-6}X_{6it}$$

Based on the individual-time effect FEM model results, the goodness of the model is 97.65 %, which means that the variables can explain 97.65 % of the MIT variable.

### E.  Best model interpretation

The best model obtained is the individual-time effect FEM model after handling the heteroscedasticity case. Based on the coefficient of determination, a value of 97.65 % is obtained, which means that the variables can explain the MIT variable of 97.65 %. Based on the best model, it is known that for Indonesia to be released from MIT or to increase GRDP per capita, it can be done by taking into account the variables that have a significant effect, namely Life Expectancy $\left(X_{2}\right)$, Gross Enrollment Rate $\left(X_{4}\right)$, and Gross Fixed Capital Increase $\left(X_{6}\right)$. The value of $\alpha_{0}$ is the intercept for each Province. While the interpretation of the model is if the variable Life Expectancy $\left(X_{2}\right)$ increases by 1 unit, the GRDP per capita will increase by US$ 402.67. If the Gross Enrollment Rate $\left(X_{4}\right)$ increases by 1 unit, the GRDP per capita will decrease by US$ 18.3, and if the Gross Fixed Capital Increase $\left(X_{6}\right)$ increases by 1 million rupiahs, then the GRDP per capita will increase by US$ 5.49 × 10^−6^.

## Conclusions

Modeling the factors affecting the Middle Income Trap in Indonesia using Panel Data Regression has been successfully carried out. Based on the analysis and discussion described, the panel data regression estimation results provide the best model, namely the FEM model of individual and time effects. This model has three variables that significantly influence MIT, namely, Life Expectancy $\left(X_{2}\right)$, Gross Enrollment Rate $\left(X_{4}\right)$, and Gross Fixed Capital Increase $\left(X_{6}\right)$, with a coefficient of determination of 97.65 % and fulfill the modeling assumptions. Based on the analysis results, suggestions for all elements involved are to pay attention to the factors that influence MIT to be used as a reference in making policies—suggestions for further research by accommodating the spatial effects of each province using spatial panel data regression.

## Ethics statements

The data used in this research are secondary data derived from the official website of Badan Pusat Statistik and World Bank.

## CRediT authorship contribution statement

**Vita Ratnasari:** Conceptualization, Methodology, Writing – original draft, Supervision. **Salsabila Hidayatul Audha:** Data curation, Formal analysis, Visualization. **Andrea Tri Rian Dani:** Data curation, Formal analysis, Visualization, Writing – review & editing.

## Declaration of Competing Interest

The authors declare that they have no known competing financial interests or personal relationships that could have appeared to influence the work reported in this paper.

## Data Availability

The authors do not have permission to share data.
